# Proteoglycan synthesis by human corneal explants submitted to laser in situ keratomileusis (LASIK)

**Published:** 2007-02-01

**Authors:** Suy Anne Rebouças Martins, Mauro Q. Campos, Benedicto C. Vidal, Alessandra G. A. Berto, Jair A. K. Aguiar, Yara M. Michelacci

**Affiliations:** 1 Departamento de Oftalmologia, Universidade Federal de São Paulo, Escola Paulista de Medicina - UNIFESP - São Paulo, SP, Brazil; 2 Departamento de Biologia Celular, Universidade de Campinas, Campinas, SP, Brazil; 3 Departamento de Bioquímica, Universidade Federal de São Paulo, Escola Paulista de Medicina - UNIFESP - São Paulo-SP, Brazil

## Abstract

**Purpose:**

To evaluate the acute effects of laser in situ keratomileusis (LASIK) upon the synthesis of proteoglycans (PGs) and collagen fibril organization in human corneal explants.

**Methods:**

Human corneas that had been rejected for transplants were obtained at Banco de Olhos of Hospital São Paulo. For each eye pair, one cornea was submitted to refractive surgery, and the other was used as its matched control. After surgery, the corneas were excised from the eyes and immediately placed in a Ham F-12 nutrient mixture containing ^35^S-sulfate for the metabolic labeling of PGs. After 24 h incubation, PGs were extracted and identified by a combination of agarose gel electrophoresis and enzymatic degradation with protease and specific glycosaminoglycan lyases. Histopathological and birefringence analysis were performed in fixed tissue slices.

**Results:**

A marked decrease in ^35^S-sulfate incorporation in PGs was observed in corneal explants that received LASIK, especially concerning dermatan sulfate-PGs, with keratan sulfate- and heparan sulfate-PG synthesis reduced to a lower degree. Only low molecular weight PGs were present in the corneas, both before and 24 h after LASIK. No sign of wound healing processes were observed, but a marked change in corneal birefringence was seen following LASIK treatment.

**Conclusions:**

Laser application led to decreased PG biosynthesis in human corneal explants, with marked changes in the collagen fibril organization, as revealed by changes in the tissue birefringence.

## Introduction

Laser in situ keratomileusis (LASIK) has become the most frequently used refractive procedure in the world [1], due to rapid visual recovery and lack of significant pain. More than one million procedures are estimated to have been performed worldwide [1,2]. This procedure represents a combination of previously used techniques in refractive surgery. It involves the use of a microkeratome to create a thin corneal flap, followed by excimer laser ablation of the corneal stroma and repositioning of the flap [2]. Corneal lamellar dissection was first described in 1949 by Barraquer [3] as part of keratomileusis surgery, and it was later modified to become an integral component of automated lamellar keratoplasty (ALK). Corneal excimer laser ablation has been used in photorefractive keratectomy (PRK) since 1983. Further advances in excimer laser technology and the development of safer microkeratomes have allowed lamellar refractive surgery to expand from a surgical technique performed by only a few experts to a widespread procedure, now performed by the general ophthalmic surgeon [2,4,5].

The clinical outcome of laser refractive surgery may be less than ideal for a group of patients because the cornea, as a biological structure, is subject to individual variation in the healing response [6]. Variability in wound healing response among patients is progressively gaining more attention because it affects the degree of prediction and stability of all refractive surgical procedures. With the development of molecular and cellular biology, the understanding of the molecular basis for this variation is becoming increasingly feasible [6,7].

The stromal cells secrete an extracellular matrix composed mainly of collagen fibrils arranged in orthogonal lamellae, and proteoglycans [8]. Maurice [9] attributed the transparency of the cornea to the regular spacing of corneal fibrils, and the corneal proteoglycans play a role in the collagen fibrilogenesis and matrix assembly. There are strong evidences suggesting the involvement of proteoglycans in the development and maintenance of corneal transparency. See reference [10] for a review on corneal collagen and proteoglycans.

Proteoglycans are macromolecules that have a protein core with covalently linked glycosaminoglycan side chains [11]. In many species, the predominant corneal glycosaminoglycans are dermatan sulfate and keratan sulfate, with smaller amounts of heparan sulfate. The main corneal extracellular matrix proteoglycans belong to the small leucine-rich family of proteins (SLRP). Some members of this family are known to regulate collagen fibrillogenesis [10]. In 1992, a cDNA clone encoding the lumican core protein of a chick corneal keratan sulfate proteoglycan [12] was obtained, and the bovine [13] and human [14] lumican core proteins were cloned later. Two other keratan sulfate proteoglycans, keratocan and mimecan (or osteoglycin), were cloned from the bovine cornea [15]. Although expressed in other tissues, lumican, keratocan, and mimecan are glycosylated only in the cornea with sulfated keratan sulfate chains. The crucial role of lumican in the regulation of collagen assembly into fibrils was established in studies on mice with bilateral corneal opacit that were homozygous for a null mutation in lumican [16]. Furthermore, it was shown that mimecan-deficient mice have thicker collagen fibrils in both corneal and skin preparations [17]. Nonglycosylated core protein of lumican is as effective as the intact proteoglycan in inhibiting fibrillogenesis in vitro [18], but the glycosylation is important to the corneal transparency process [19].

The main chick corneal dermatan sulfate proteoglycan was identified as decorin [20]. It was shown that synthesis of nonglycosylated decorin in avian cornea leads to disruption in lamellar organization, suggesting that dermatan sulfate proteoglycans are not involved in the regulation of collagen fibril diameter, but are important to fibril-fibril spacing and lamellar cohesiveness [21]. Furthermore, in corneal explants from embryonic chicken, an increased synthesis of keratan sulfate proteoglycan and a decreased synthesis of dermatan sulfate proteoglycan coincided with the onset of tissue transparency, again suggesting a correlation between proteoglycan composition and corneal transparency [21].

The main heparan sulfate proteoglycans found in cornea are perlecan [22], a basement membrane proteoglycan, and syndecan, a cell surface proteoglycan expressed mainly by epithelial cells [23,24].

The aim of the present study was to evaluate the effects of LASIK on proteoglycan biosynthesis and collagen organization in human cornea explants, in comparison to paired controls.

## Methods

### Materials

Keratanase (from *Pseudomonas* species) [25], Ham F-12 culture medium, fetal bovine serum, and papain were purchased from Sigma Chemical Co., Inc. (St. Louis, MO). Chondroitinases AC and B, and heparitinase II (from *Flavobacterium heparinum*) were prepared by methods previously described in reference [26]. Agarose (standard, low M_r_) was purchased from Bio-Rad Laboratories (Richmond, CA). Acrylamide, N,N-methylenebisacrylamide, N,N,N',N'-tetramethylenediamine, 1,3-diaminopropane, and ethylenediamine were from Aldrich Chemical Co., Inc. (Milwaukee, WI). H_2_^35^SO_4_ (carrier free) was purchased from Instituto de Energia Nuclear (São Paulo, SP, Brazil). Ultima Gold liquid scintillation cocktail was purchased from Packard Instruments Co., Inc. (Meriden, CT), and 2,5-diphenyloxazole (PPO) was from Beckman Instruments, Inc. (Fullerton, CA).

### Tissue Source

We obtained 24 human corneoscleral buttons from 12 donors, aged between 20 and 75 years, from Banco de Olhos do Hospital São Paulo (São Paulo, SP, Brazil). Only eyes from donors who provided specific consent for research purposes and with a contraindication for transplantation were included. The eyes were stored at 4 °C for a maximum of 48 h postmortem, in a wet chamber containing 4 mg/ml gentamycin in phosphate buffered saline (PBS). Detailed information about characteristics of the donors and corneal specimens is provided in Table 1.

**Table 1:** Demographics of LASIK and control corneas.

**Cornea samples (pairs)**	**Cornea type**	Location* (OD/OS)	**Donor (Age (y)/Gender)**	**Time interval (Death-LASIK)**
1	LASIK	OS	61/F	6 h
	Control	OD	61/F	6 h
2	LASIK	OS	58/M	8 h
	Control	OD	58/M	8 h
3	LASIK	OS	20/M	8 h
	Control	OD	20/M	8 h
4	LASIK	OS	47/M	10 h
	Control	OD	47/M	10 h
5	LASIK	OS	53/M	6 h
	Control	OD	53/M	6 h
6	LASIK	OS	74/M	12 h
	Control	OD	74/M	12 h
7	LASIK	OS	72/F	24 h
	Control	OD	72/F	24 h
8	LASIK	OS	66/F	48 h
	Control	OD	66/F	48 h
9	LASIK	OS	68/F	8 h
	Control	OD	68/F	8 h
10	LASIK	OS	75/M	10 h
	Control	OD	75/M	10 h
11	LASIK	OS	62/M	10 h
	Control	OD	62/M	10 h
12	LASIK	OS	64/M	10 h
	Control	OD	64/M	10 h

LASIK was performed in 12 corneoscleral buttons from 12 donors. The 12 contralateral corneoscleral buttons were kept intact and used as controls.

All 24 corneoscleral specimens (LASIK [n=12]; controls [n=12]) were evaluated for gross abnormalities with a slit lamp (BP 900; Haag Streit, Switzerland), using direct and indirect illumination. None of the 12 donors had any history of corneal surgery, and no corneal pathologies were found.

Methods for obtaining and handling of human tissue were humane, and the tenets of the Declaration of Helsinki were followed. Informed consent was obtained from each donor's family after explanation of the nature and possible consequences of the study.

### Surgical technique: LASIK

LASIK is a two-step surgical technique for the performance of a refractive corneal surgery. The first step consists of using the microkeratome to create a partial thickness corneal flap. The microkeratome consists of two components: a ring, which is applied to the globe and can harden it by creating a vacuum, and an oscillating blade, which fits onto the vacuum ring and cuts a partial thickness corneal flap. The cut is incomplete, leaving a small hinge, which allows the surgeon to lift the flap, treat the corneal stroma with the excimer laser, and replace the flap without suturing at its original location [2]. The second step uses the excimer laser, in the same manner as in PRK.

Excimer laser photoablation was performed with a flying-spot laser (LADAR Vision® System; Summit Autonomous Technologies, Orlando, FL), with repetition rate of approximately 55 pulses per second, a spot size of 0.8-0.9 mm, an average fluence of 180-240 mJ/cm^2^, and a pulse energy of 2.4-3.0 mJ. The corneas received a 6.0 mm ablation zone diameter, 6.0 diopter myopic correction to a theoretical stromal ablation depth of 68.3 mm. All surgeries were performed by the same surgeon. For each eye pair, one cornea was submitted to LASIK, and the other was used as a matched control.

LASIK was performed in 12 corneas from 12 donors. The ocular globes were secured in an ocular globe holder. A hinged flap was created with a microkeratome (Hansatome; B&L, Rochester, NY) with a 160 head and 8.5 ring, and excimer laser photoablation was immediately performed with a flying spot laser (Summit Autonomous Technologies, LADAR Vision® System). The stromal bed was then irrigated with saline solution, and finally the flap was folded back onto the cornea. The other 12 contralateral eyes were kept intact and used as controls.

### Tissue culture and radioisotope labeling of corneas

After the surgery, the corneas were excised under sterile conditions from the eyes, leaving 1.5 mm scleral rims preserved. The corneas were then washed with 5 ml of a 4 mg/ml gentamycin solution in PBS, and immediately placed in 12 ml of HAM F-12 nutrient mixture supplemented with 10.000 U of penicillin and 100 mg of streptomycin, containing 100 mCi/ml ^35^S-sulfate for the metabolic labeling of proteoglycans. The corneas were labeled with ^35^S-sulfate for 24 h at 37 °C in 2.5% CO_2_ atmosphere. After ^35^S-sulfate incorporation, the corneas were trimmed free of the scleral rims, and the proteoglycans were extract and analyzed. The peripheral cornea surrounding the LASIK scar was included in the proteoglycan measurement.

### Extraction, identification, and quantification of proteoglycans and glycosaminoglycans

After 24 h incubation, the corneal explants were halved. One half was used for proteoglycan extraction, and the other was used for histophatological and birefringence analysis. For proteolgycan extraction, the corneal explant fragments were weighed, cut in small pieces (<0.5 mm), and suspended in 4 M guanidine hydrochloride (GuHCl) in 0.5 sodium acetate buffer, pH 5.8. After overnight incubation at 4 °C with shaking, debris was removed by centrifugation and the proteoglycans were precipitated by the addition of ethanol (3 volumes) to the supernatant. Subsequently, the precipitates formed were collected by centrifugation, washed with 80% ethanol, and dried. The dried material was resuspended in 100 μl of distilled water and analyzed for proteoglycans by a combination of agarose gel electrophoresis and enzymatic degradation with specific glycosaminoglycan lyases, using a technique described in reference [27]. For the analysis of glycosaminoglycans, the proteoglycans were submitted to proteolysis with papain (2 mg/ml in 0.06 M phosphate-cysteine buffer, pH 6.5, containing 20 mM EDTA-1 ml of solution per 100 mg of tissue wet weight) using a previously described technique [27,28]. The proteoglycans and their degradation products were fixed on the gel with cetyltrimethylammonium bromide and stained with toluidine blue. The ^35^S-sulfate labeled compounds were visualized by exposure of the agarose gel slabs (after fixation, drying, and staining) to a Packard Cyclone^TM^ Storage Phosphor System by 1-3 days. For quantification, they were scraped off the agarose gels and counted in a Beckman 6800 liquid scintillation spectrophotometer, using Ultima Gold LSC-Cocktail [29]. The quantitative results were always corrected for ^35^S-decay.

### Histopathology and birefringence microscopy

Corneal halves were fixed in paraformaldehyde (paraformaldehyde in 0.05 M phosphate-buffered saline-PBS, pH 7.4) for 4 h at room temperature and then rinsed in phosphate buffer. The corneas were dehydrated in graded alcohol washes and embedded in paraffin. Ultra-thin sections were cut on a microtome (5 μm) and stained with hematoxylin-eosin (H&E) for standard morphology [30].

For fluorescence microscopy with DAPI, the fixed tissue fragments were rinsed with PBS, embedded in tissue freezing medium, and stored at -20 °C. Cryostat cross-sections were taken from the corneal central area, air dried and stained with DAPI (1:1000 dilution in PBS) for 5 min to localize cell nuclei. Sections were analyzed under conventional fluorescence microscopy or confocal microscopy [31].

For birefringence analysis, 8 μm sections of human corneal buttons were used after rehydration. All observations were carried out with transmitted polarized light using a Zeiss microscope (40X). The optical retardation was measured by means of a quarter-wave plate at a wavelength of 546 μm [32]. The corneas were examined with polarized light microscopy 24 h after LASIK. The Sènarmont compensator is connected to the light microscope, so the anisotropic specimen (the human cornea) is oriented in a subtractive position that at some tilt angle extinction occurs, provided the compensator's range is sufficient for the sample. Compensators consist of thin-sections of minerals (e.g., quartz, calcite, gypsum/selenite, mica, etc.), or polymer film equivalents, whose thickness and optical orientation are carefully controlled so as to provide known values of retardation and known direction of high and low refractive indices. When introduced into the light path of a polarizing microscope, these known retardation values and refractive index directions are superimposed on an unknown anisotropic specimen and, by noting the resultant effects (addition or subtraction of retardation, etc.), give valuable identifying characteristics of the sample. The specific axis and magnitude of the cornea collagen fibrils is determined by first imaging the cornea without compensation. However, in uncompensated scans, a nonuniform retardation pattern is present. The statistical significance was calculated using analysis of variance between groups (ANOVA).

## Results

**Figure 1:**
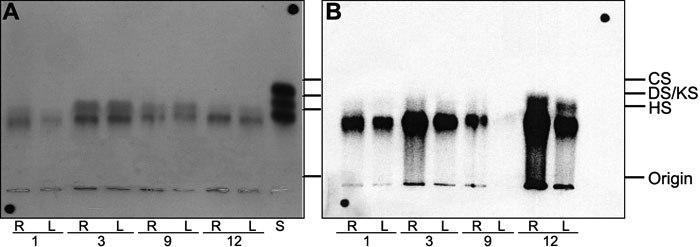
Agarose gel electrophoresis of proteoglycans extracted from human corneal explants. The left (L) cornea of each pair was submitted to LASIK, and the right (R) cornea was its matched control. The corneal explants were maintained under tissue culture conditions for 24 h in the presence of ^35^S-sulfate for the metabolic labeling of proteoglycans. The proteoglycans were extracted as described in Methods, and aliquots (5 μl) were submitted to agarose gel electrophoresis, as also described in Methods. The proteoglycans were fixed in the gel and stained by toluidine blue (**A**), and the ^35^S-labeled proteoglycans were localized by radioautography (**B**). In the images, S indicates a mixture of standard glycosaminoglycans containing chondroitin sulfate (CS), dermatan sulfate (DS), and heparan sulfate (HS), 5 μg each. KS indicates keratan sulfate. The numbers 1, 3, 9, and 12 refer to the corneal pairs described in Table 1.

Figure 1 shows a representative electrophoresis of proteoglycans extracted from control (right, R) and LASIK-treated (left, L) corneal explants, stained with toluidine blue (total proteoglycans) and detected by radioautography of the agarose gel slabs (metabolically labeled ^35^S-proteoglycans, synthesized during the last 24 h). The main labeled band, observed in all samples, migrated slightly less than the standard heparan sulfate. A minor band of faster migration was observed in some samples (2 and 4 in Figure 1). The total amounts of proteoglycans extracted for each cornea pair were roughly the same, based on toluidine blue staining, indicating that no variations occurred in the extraction yields. The ^35^S-labeling was found to be greatly decreased in LASIK-treated corneas (see radioautogram, "R" versus "L" for each pair).

**Figure 2:**
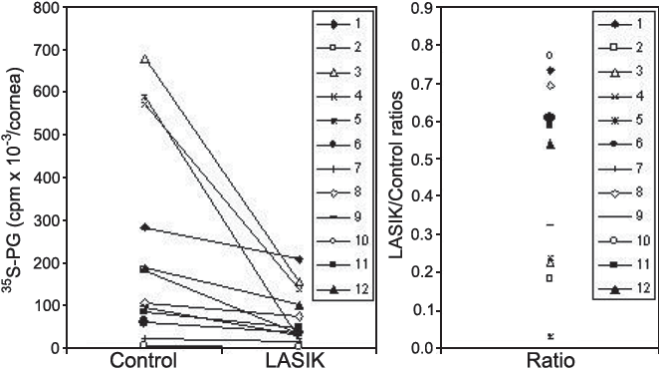
Effect of LASIK upon proteoglycan synthesis by human corneal explants. The experiments were performed as described in Figure 1, except that individual data are presented. The numbers in the insert box refer to the corneal pairs described in Table 1, and each point is the mean value of three determinations.

Figure 2 shows the individual paired results. A decrease in ^35^S-sulfate incorporation in proteoglycans was observed in all cases after LASIK.

In order to check if the biosynthesis of all proteoglycans was affected, the glycosaminoglycan chains were released from the protein cores by proteolysis. They were identified by a combination of agarose gel electrophoresis and enzymatic degradation with specific glycosaminoglycan lyases, as described in Methods.

**Figure 3:**
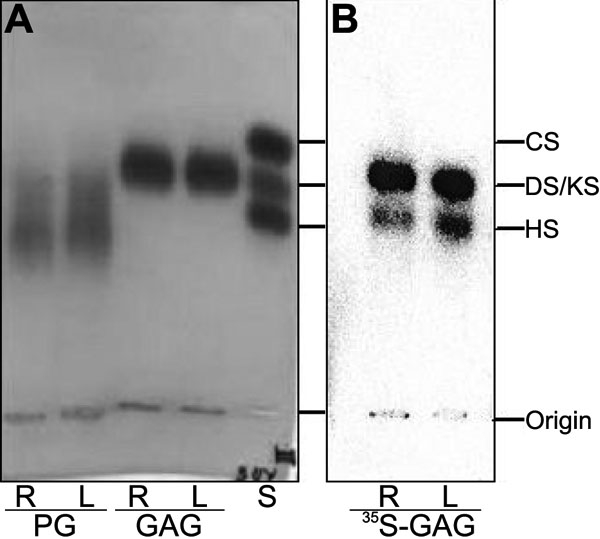
Agarose gel electrophoresis of glycosaminoglycans released by proteolysis from proteoglycans extracted from human corneal explants after LASIK. The proteoglycans extracted from pair number 12 (see Table 1) of human corneal explants underwent proteolysis with papain for release of the glycosaminoglycan chains. Aliquots of the incubation mixtures were submitted to agarose gel electrophoresis. Total proteoglycans (PG) and glycosaminoglycans (GAG) were stained by toluidine blue (**A**), and the ^35^S-labeled glycosaminoglycans (^35^S-GAG) were localized by radioautography (**B**). S: A mixture of standard glycosaminoglycans containing chondroitin sulfate (CS) dermatan sulfate (DS), and heparan sulfate (HS), 5 μg each; KS, keratan sulfate; PG, intact proteoglycans.

The electrophoretic migration of the glycosaminoglycan chains released from the core proteins by proteolysis is shown in Figure 3. Upon incubation of the proteoglycans with papain, the band(s) corresponding to proteoglycans completely disappeared, and one band, migrating as dermatan sulfate and keratan sulfate, appeared upon toluidine blue staining. Nevertheless, on radioautogram, a second band, migrating as heparan sulfate, could also be detected. Chondroitin sulfate was not found.

**Figure 4:**
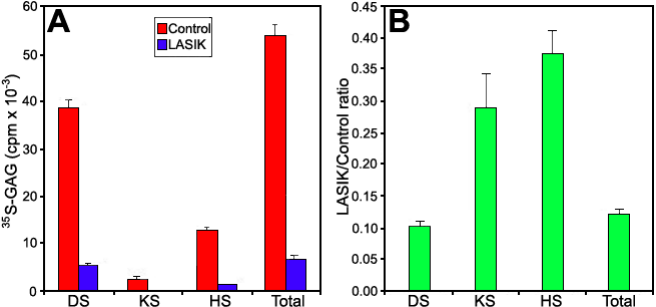
Effect of LASIK upon the synthesis of ^35^S-dermatan sulfate, ^35^S-keratan sulfate, and ^35^S-heparan sulfate by human corneal explants. Shown are the ^35^S-sulfate incorporation (**A**; cpm, mean±standard error, three determinations for each sample) and the ratio between LASIK and control (**B**). The identification of each glycosaminoglycan was based on a combination of agarose gel electrophoresis and enzymatic degradation with specific glycosaminoglycan lyases (chondroitinases, keratanase and heparitinase).

To confirm the glycosaminoglycan identification, these compounds were incubated with specific glycosaminoglycan lyases. Upon the action of *F. heparinum* chondroitinase B, dermatan sulfate was degraded to oligosaccharides and disaccharides that were not fixed in the gel [33]. The band migrating as heparan sulfate disappeared upon the action of heparitinase II, and upon the action of keratanase, keratan sulfate was digested. *F. heparinum* chondroitinase AC had no effect upon corneal glycosaminoglycan, confirming that chondroitin sulfate was not present and corneal dermatan sulfate is predominantly a L-iduronic acid containing polymer. Figure 4 shows the mean±standard error of all the samples (three determinations for each sample), obtained after release of the glycosaminoglycan chains by proteolysis of the ^35^S-labeled proteoglycans. The synthesis of all glycosaminoglycans decreased after LASIK, but the synthesis of dermatan sulfate decreased more, leading to a decrease in the relative proportion of this glycosaminoglycan. The degradation products formed after the action of the glycosaminoglycan lyases were the same for LASIK-treated and control corneas, indicating that this decrease in ^35^S-sulfate incorporation is not due to the synthesis of undersulfated proteoglycans.

**Figure 5:**
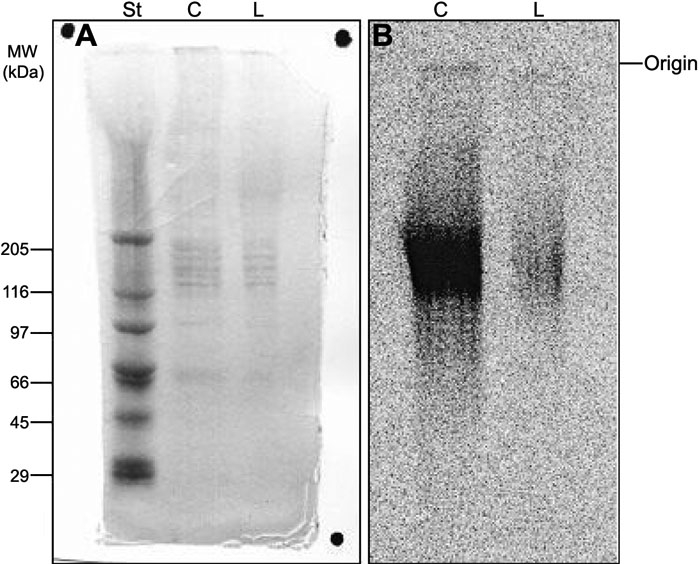
Polyacrylamide gel electrophoresis of proteoglycans extracted from human corneal explants. Aliquots (5 μl) of proteoglycans extracted from pair number 1 (see Table 1) of human corneal explants were submitted to polyacrylamide gel electrophoresis, as described in Methods. The gel was stained with coomassie blue (**A**), and the ^35^S-labeled compounds were localized by radioautography (**B**). St: molecular weight standard proteins; C: control cornea; L: LASIK submitted cornea.

Figure 5 shows a representative polyacrylamide gel electrophoresis of the proteoglycans [34] extracted from human corneal explants after LASIK. This analysis was performed for all samples. It showed that only low molecular weight proteoglycans (probably of the decorin/biglycan and lumican/mimecan families) are present in human cornea, both before and after LASIK.

**Figure 6:**
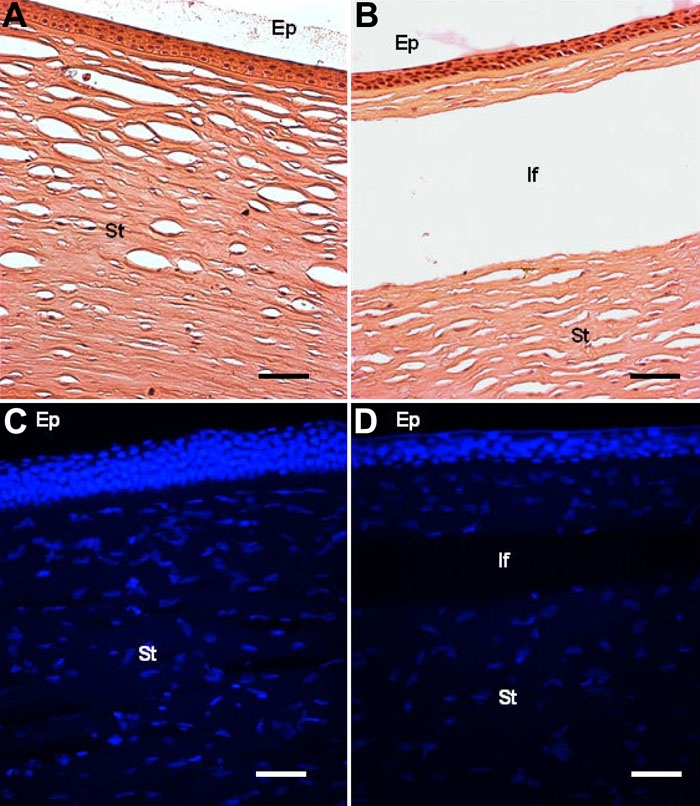
Hematoxilin and eosin staining and fluorescence microscopy for nuclei (DAPI) of normal and LASIK-treated corneal explants. The experiment was performed as described in Methods. Ep: epithelium; If: interface after LASIK; St: stroma. The scale bar equals 50 μm.

Figure 6 shows representative images of H&E and DAPI staining of corneas submitted to LASIK Figure 6B,D and their respective controls Figure 6A,C. H&E analysis did not show any histological alteration after LASIK procedure. DAPI staining showed a reduction in the number of cell nuclei seems to occur after LASIK (quantitative data not yet available).

**Figure 7:**
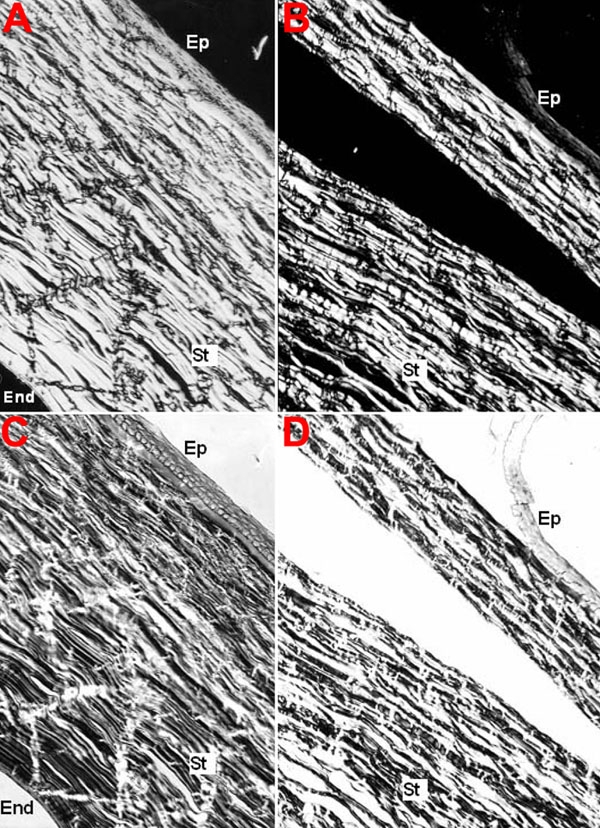
Control and LASIK human cornea birefringence images. The experiment was performed as describe in Methods, with (**C**,**D**) and without (**A**,**B**) compensation, for control (**A**,**C**) and LASIK-submitted (**B**,**D**) corneal slices. The samples are positioned 45° from the polarizer azimuth. Ep: epithelium; St: stroma; End: endothelium. The scale bar is equal to 20 μm.

Figure 7 illustrates typical birefringence images of a normal (Figure 7A,C) and a LASIK-treated cornea (Figure 7B,D), with dark and bright shadows and also some visible irregularities in the intensity. The corneal collagen fibrils were oriented 45° from the polarizer azimuths, allowing maximum shining of the fibrils. Sample Figure 7A represents the control cornea section without compensation. In this human corneal section, collagen fibrils are seen as either parallel aligned linear structures or as hexagonal arranged structures. Figure 7C represents the same corneal micrograph with compensation to allow the measurements of optical retardation values. In birefringence images of human corneal sections after LASIK (Figure 7B,D), only minor differences were observed in collagen fibril alignments when compared to the control cornea. These differences are evident after obtaining statistical analysis of the quantitative data of optical retardation (Table 2). Eighty-two measurements were performed for each corneal group (Control and LASIK) in different regions of corneal slices. Note that there is a significant decrease in optical values after LASIK, in comparison to the control group.

**Table 2:** Optical retardation mean values for control cornea and cornea submitted to LASIK, embedded in water.

**Cornea samples**	**N**	**OR**	**p**
Control	82	24.96	<0.0001
LASIK	82	17.92	

Statistical significance was calculated by analysis of variance between groups (ANOVA), as described in the text. Eighty-two measurements were performed for each corneal group (Control and LASIK) in different regions of corneal slices. In the table, "N" indicates the number of measurements for each group, "OR" indicates the optical retardation value, and "p" denotes the significance level.

## Discussion

The relevance of the regular diameter and distribution of collagen fibrils in stroma for corneal transparency is well documented [9] as well as the effects of proteoglycans of the SLRP or "fibrillar" family upon the collagen fibrilogenesis and extracellular matrix assembly (review in [10]). This is especially important in processes, such as corneal wound healing, that could lead to corneal haze and opacity, affecting both tissue transparency and biomechanics. These are serious concerns in refractive surgery. The organization of collagen fibrils in corneal stroma may be assessed by birefringence studies [35-39], and the proteoglycans present are usually identified by immunohistochemical studies [40]. Nevertheless, these latter studies localize all the proteoglycans present in the tissue - not just those synthesized after the surgical procedures - and do not permit the structural characterization of proteoglycans.

Biochemical analyses of human corneas after LASIK are rare [41]. Most studies have been performed in experimental animals [42-44]. The main proteoglycans of human corneal stroma are keratan sulfate and dermatan sulfate [25,29,45]. In our study, the distribution of these compounds was only slightly altered several months after LASIK [46]. Quantock et al. [47] showed that sulfated proteoglycans exist in rabbit corneas healing from lamellar incisions one, two, and three weeks after the surgical procedure. A recent clinico-pathological study of two human corneas that had received successful LASIK treatment reported an active wound-healing response at the flap-bed interface with identification of periodic acid Schiff-positive, electron-dense, material three months postoperative [48].

Our study used human corneas because morphological investigations have shown that, when this tissue is treated in vitro with LASIK and then maintained in culture for different periods, there are striking clinical similarities in LASIK-treated human and rabbit eyes [49]. As the inflammatory response after LASIK is low and no inflammatory cells usually enter the cornea, the in vivo situation is likely to be independent of systemic factors that are absent in vitro [50,51].

Although several groups have studied the glycosaminoglycan composition of cornea as well as the biosynthesis of proteoglycans by rabbit and embryonic chicken corneal explants [10,12,20], there are few reports concerning human corneal proteoglycans [14,25,29]. The aim of our study was to investigate the effects of LASIK upon proteoglycan synthesis by corneal human explants.

We found all human corneal explants were able to incorporate ^35^S-sulfate in proteoglycans, but variations occurred in the biosynthesis rate among different corneal pairs. These variations could not be attributed to single features, such as donor age or time period between donor death and experiments, but are possibly due to the combined interference of multiple biological conditions.

Nevertheless, in all cases reported here, a marked decrease in ^35^S-sulfate incorporation in proteoglycans occurred during the first 24 h after LASIK, in comparison to their respective matched controls. This could be due, at least in part, to the synthesis of undersulfated proteoglycans, but structural analysis with specific glycosaminoglycan lyases revealed no changes in the sulfation degree. This decrease in label incorporation possibly reflects a decrease in the proteoglycan synthesis rate.

The synthesis of all proteoglycans was disturbed, but dermatan sulfate proteoglycans were the most strongly affected. This is not surprising, since the main proteoglycans synthesized in a 24 h period by human corneal explants are dermatan sulfate proteoglycans [25,29]. Keratan sulfate proteoglycans are synthesized at lower rates, and heparan sulfate proteoglycans are minor components of the cornea. Moreover, only low molecular weight proteoglycans were detected. High molecular weight proteoglycans, such as versican (which could be synthesized as a healing response), could not be detected.

This conspicuous decrease in proteoglycan synthesis could be due to epithelial lesion induced by the lamellar cut created by the microkeratome as well as to stromal photoablation with excimer laser. However, the 50-60-μm-thick region ablated by LASIK from the 6 mm central area of the cornea corresponds to about 4% of total stromal tissue, and the decrease in ^35^S-sulfate incorporation in proteoglycans was close to 90%. This clearly indicates that the ablated tissue could not be the only factor responsible for the enormous decrease in proteoglycan synthesis reported here. Keratocyte cell death could possibly be also implicated.

Light microscopy did not reveal any traces of corneal wound healing. Again, this is not surprising since this is an acute in vitro study. Beneath the flap, corneal collagen bundles seemed to be uniform and regular, but upon birefringence studies, some degree of fiber dearrangements could be observed. There was a decrease in optical retardation values following LASIK, leading to a decreased molecular corneal organization, that may be related to the conspicuous changes in proteoglycan synthesis.

In conclusion, the data here presented indicate a marked decrease in proteoglycan synthesis and changes in corneal collagen organization 24 h after in vitro LASIK surgery in human cornea.
